# An atypical and diagnostically challenging case of pediatric metastatic Crohn’s disease

**DOI:** 10.1016/j.jdcr.2026.03.022

**Published:** 2026-03-19

**Authors:** Inès Belkasmi, Olivier Philip, Anne Jouvenet, Benoit Catteau, Sarah Faiz, Yoan Ditchi, Chloé Vuillamy

**Affiliations:** a Dermatology, Douai Hospital, Douai, France; b Pediatric Dermatology, CHU Lille, Lille, France; c Pediatrics, Douai Hospital, Douai, France; d Dermatopathology, Unilabs Lille Pathology Laboratory, Lille, France

**Keywords:** cutaneous granuloma, cutaneous manifestation of Crohn disease, gastro-enterology, metastatic Crohn's disease, paradoxical reactions of anti TNF alpha, pathology, pediatric metastatic Crohn's disease, pediatrics

## Introduction

We report a case with both clinically and histopathologically atypical presentation of pediatric metastatic Crohn's disease presenting as an erythematous-violaceous maculopapular rash mimicking Sweet's syndrome. This case highlights the importance of multidisciplinary consultation among dermatologists, gastroenterologists, and pathologists.

## Case

**Fig 1:**
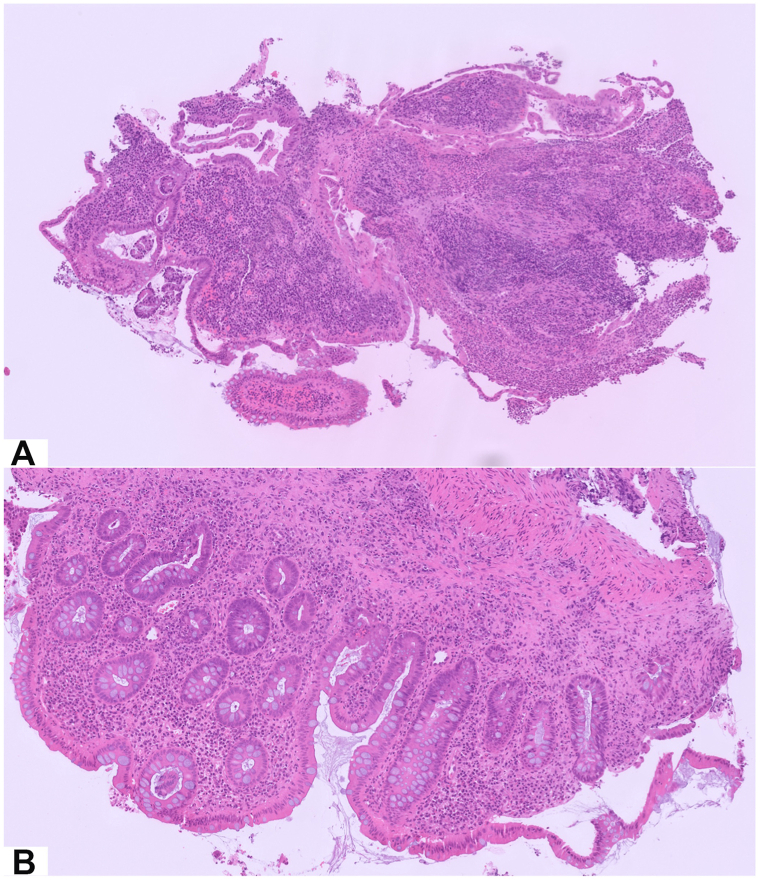
Digestive histologic features **(A)** Ileal biopsy, Hematoxylin and Eosin (HE) stain, ×5 **(B)** Colon biopsy HE stain, ×10.

We report the case of a 12-year-old girl followed by pediatric gastroenterologists for Crohn’s disease, diagnosed in early 2025 after presenting with chronic diarrhea and significant weight loss. The diagnosis of Crohn’s disease was made anatomo-clinically: endoscopic intestinal biopsies (Fig 1) showed ulcerative ileitis presenting with architectural disorganization and polymorphic infiltrate of the chorion (Fig 1, *A*), as well as acute colonic inflammation characterized by cryptitis lesions associated with chronic remodeling (Fig 1, *B*) suggestive of inflammatory bowel disease, without granuloma. After the failure of exclusive enteral nutrition with Modulen, adalimumab (Yuflyma) 20 mg every 2 weeks was initiated on July 2, 2025, in combination with a progressively tapered course of oral corticosteroids (Solupred 30 mg).

**Fig 2:**
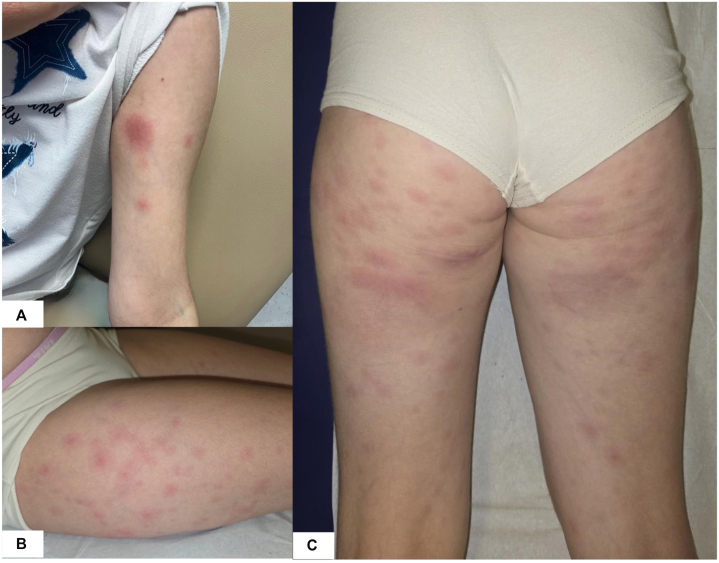
Cutaneous metastatic Crohn’s disease: clinical cutaneous presentation. **A,** Arm, **(B)** lateral surface of the right leg, and **(C)** back of the legs.

The fourth injection of adalimumab was administered on August 14, 2025. Within 24 hours, the patient developed fever, a flare of Crohn’s disease, and diffuse, non-tender erythematous nodules predominantly affecting the arms and legs, occurring in 3 successive episodes (Fig 2). Her general condition remained preserved, and physical examination showed no further abnormalities, including the absence of mucosal involvement.

Laboratory investigations demonstrated a marked inflammatory syndrome with leukocytosis (19 × 10^9^/L) and an elevated C-reactive protein level of 168 mg/L. Serologic investigations were negative for HIV, HBV, HCV, and rickettsial infection. EBV and Parvovirus B19 results were consistent with past infection. Mycoplasma IgM was detected at the threshold level without associated clinical signs. Based on this presentation, a Sweet’s syndrome was initially suspected. The corticosteroid taper was therefore reversed, and the dose increased to 20 mg/day (0.5 mg/kg/d), leading to a rapid regression of the cutaneous lesions.

An initial skin biopsy revealed a dermal annular granuloma, which was first interpreted as a paradoxical reaction to adalimumab. PAS and Ziehl-Neelsen staining did not reveal any pathogens.

During subsequent gastroenterology follow-up, the patient developed secondary loss of response to adalimumab, with end-of-dose symptoms and persistently subtherapeutic trough levels (<1 μg/mL), indicating insufficient drug exposure and ongoing disease activity. These findings prompted a reassessment of the initial diagnosis, as a paradoxical reaction was unlikely in the context of treatment inefficacy.

**Fig 3:**
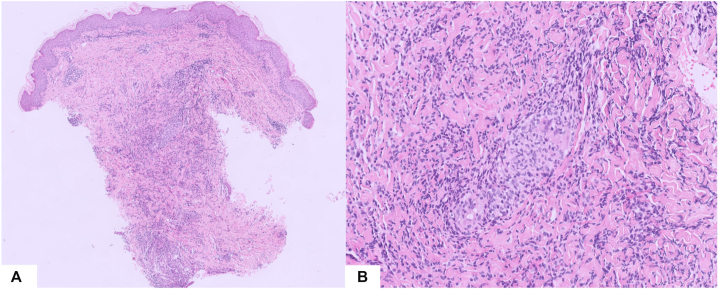
Cutaneous histologic features **(A)** ×5 Hematoxylin–Eosin–Saffron (HES) stain, **(B)** ×20. HES stain.

A review of the initial cutaneous histopathology slides was therefore requested. Recuts were made and revealed multinucleated giant cell granulomas (Fig 3), consistent with cutaneous localizations of Crohn’s disease. As both intestinal and extra-intestinal manifestations of Crohn's disease remained uncontrolled, the adalimumab dosage was increased. The patient showed improvement in intestinal symptoms and no recurrence of cutaneous manifestations was observed.

## Discussion

Crohn's disease is a chronic inflammatory bowel disease with a relapsing-remitting course, mediated by genetic, environmental, epithelial, microbial, and immune factors with TNF-alpha as a central driver.^1^ Cutaneous manifestations represent the most frequent extra-intestinal features of Crohn's disease, occurring in over 17% of patients^1^ and in approximately 7% to 24% of pediatric Crohn’s disease cases.^2^ McKay et al reported that, in pediatric cases, the genital area was the most commonly affected site (75% of cases in their series),^3^ followed by facial involvement. Some cases describe distant cutaneous manifestations, such as pseudo-necrotic papulovesicles^4^ or a diffuse erythematous papular rash.^5^ Papulonodular manifestations, as in our patient, are rare and account for fewer than 10% of cases.^3^ Granuloma annulare also has been described as an extra-intestinal manifestation of Crohn's disease, typically presenting as annular clusters of erythematous papules.^6^ Histologically, cutaneous Crohn’s disease exhibits features similar to those of intestinal involvement, including the presence of noncaseating granulomas with foreign body-type or Langerhans-type giant cells, epithelioid histiocytes, and plasma cells.^2^

Paradoxical reactions to anti-TNFα therapies are diverse. Psoriasiform lesions are the most typical, but other granulomatous manifestations—such as sarcoidosis, interstitial granulomatous dermatitis, or granuloma annulare, as previously mentioned—have also been reported.^7^ These reactions may be explained in granulomatous reactions either by the emergence of triggering infectious agents in an immunosuppressed context or by an increase in interferon-gamma related to TNFα blockade. Histologically, the lesions may show noncaseating epithelioid granulomas or a diffuse dermal inflammatory infiltrate composed of histiocytes and neutrophils arranged in a palisading configuration around zones of collagen degeneration—an appearance closely resembling that observed in cutaneous Crohn’s disease.^7^

This case illustrates the diagnostic challenges of cutaneous Crohn’s disease mimicking Sweet’s syndrome, an atypical manifestation in metastatic Crohn’s disease. It was the worsening of digestive symptoms that led to a pathologic review, allowing the initial dermatologic diagnosis to be revised. This diagnostic reorientation reinforced the suspicion of therapeutic resistance, justifying an increase in anti-TNFα doses — a treatment that would have been reconsidered if a paradoxical reaction had ultimately been retained. The discordance between intestinal and cutaneous histology further complicates diagnosis, especially when evaluating granulomatous skin lesions in patients without granulomas in intestinal biopsies.

Granulomas are observed in 43% of pediatric digestive Crohn's disease biopsies.^8^ Although the prevalence of skin manifestations based on the presence or absence of granulomas on intestinal histopathology is poorly documented, Hong et al reported a 2.15-fold higher proportion of perineal involvement in patients with digestive granulomas.^9^ On the contrary, in the pediatric cohort described by Rothschild et al, extra-digestive manifestations—mostly arthritis—were more common in patients without granulomas at diagnosis, which is consistent with our observation.^10^ These findings open up an interesting reflection concerning the role of intestinal granulomas in the incidence of cutaneous manifestations specifically.

## Conflicts of interest

None disclosed.
